# Effects of Diet and Exercise on Mitochondrial Health in Metabolic Dysfunction-Associated Steatotic Liver Disease (MASLD): Role of Ceramides

**DOI:** 10.3390/nu17182972

**Published:** 2025-09-16

**Authors:** Jonas M. McCaffrey, Jamal A. Ibdah

**Affiliations:** 1 School of Medicine, University of Missouri, Columbia, MO 65212, USA; 2 Division of Gastroenterology and Hepatology, University of Missouri, Columbia, MO 65212, USA; 3 Harry S. Truman Memorial Veterans Medical Center, Columbia, MO 65201, USA; 4 Department of Medical Pharmacology and Physiology, University of Missouri, Columbia, MO 65212, USA; 5 Department of Nutrition and Exercise Physiology, University of Missouri, Columbia, MO 65212, USA

**Keywords:** liver, ceramides, mitochondrial dysfunction, diet, exercise, MASLD

## Abstract

Metabolic dysfunction-associated steatotic liver disease (MASLD) impacts nearly a quarter of the world’s population and encompasses a range of disease states, from simple steatosis to more advanced stages like metabolic dysfunction-associated steatohepatitis (MASH), fibrosis, and cirrhosis. A key driver of disease progression is mitochondrial dysfunction, characterized by impaired fatty acid oxidation and an overall decline in mitochondrial health. Emerging evidence has implicated ceramides—bioactive sphingolipids that serve roles in apoptotic pathways and as signals of nutrient excess—as important contributors to this dysfunction. Ceramide accumulation within mitochondria mirrors impairments seen in MASLD, specifically elevations in oxidative stress, disrupted fatty acid oxidation, and impaired mitochondrial dynamics. Ceramides may serve as an important molecular link between nutrient overload and mitochondrial dysfunction in the pathogenesis of MASLD. Given the limited availability of effective pharmacologic therapies for MASLD, lifestyle interventions like dietary modification and physical activity remain the cornerstone of disease management worldwide. In this review, we provide an overview of the current understanding of the role of ceramides in mediating the effects of diet and exercise on MASLD through their influence on mitochondrial health.

## 1. Introduction

Metabolic dysfunction-associated steatotic liver disease (MASLD) is defined by an excess of intrahepatic triglycerides and can progress to a more severe form, metabolic dysfunction-associated steatohepatitis (MASH), if left untreated [1]. MASH is heavily linked to liver-related morbidity and is histologically characterized by the presence of steatosis, lobular inflammation, and hepatocyte ballooning, with or without fibrosis [2]. Nearly a quarter of the world’s population today has characteristics indicative of MASLD, and this number will likely keep increasing alongside rising rates of obesity, insulin resistance, and sedentary lifestyles [3,4]. A major contributor to the rise in MASLD and related metabolic conditions is poor dietary habits and lack of physical activity. While pharmacologic treatments are in development, lifestyle interventions, such as diet and exercise, remain the most effective and well-proven treatments for MASLD.

A well-established hallmark of MASLD is mitochondrial dysfunction, with impaired mitochondrial fatty acid oxidation and reduced organelle quality playing major roles in the development of the disease. The modification of mitochondrial function has been shown to modulate MASLD as well [5,6]. At the cellular level, mitochondria are key components of hepatic metabolism and facilitate energy production, apoptosis, and lipid oxidation. Ceramides are a type of sphingolipid that function as signaling molecules in cell death and differentiation. Ceramides act as signals of nutrient excess and, in response to this state, accumulate and can contribute to mitochondrial dysfunction. Thus, the relationship between ceramides and mitochondria has drawn recent attention in the study of MASLD. The association between ceramides and mitochondria is even more direct, as ceramide synthase activities were recently discovered in both the outer and inner mitochondrial membranes [7,8]. Increased concentrations of ceramides in human tissues and plasma are associated with an increased risk of MASLD, as well as cardiovascular events and obesity [9,10,11]. The mechanistic effects of ceramides in mitochondria culminate in several ways that contribute to MASLD, including decreased respiratory capacity and increased apoptosis [12,13,14,15,16]. The aim of this review is to discuss the current knowledge on the relationship between mitochondria and ceramides in the context of MASLD and how lifestyle interventions of diet and exercise may impact this relationship.

## 2. The Relationship Between Mitochondria and Ceramides

Complementary studies in both humans and rodent models have highlighted the different species of ceramides, their cellular distributions, signaling functions, and vital relationships with the mitochondrial organelle, as reviewed herein.

### 2.1. Ceramide Synthesis, Degradation, and Species

**Figure 1:**
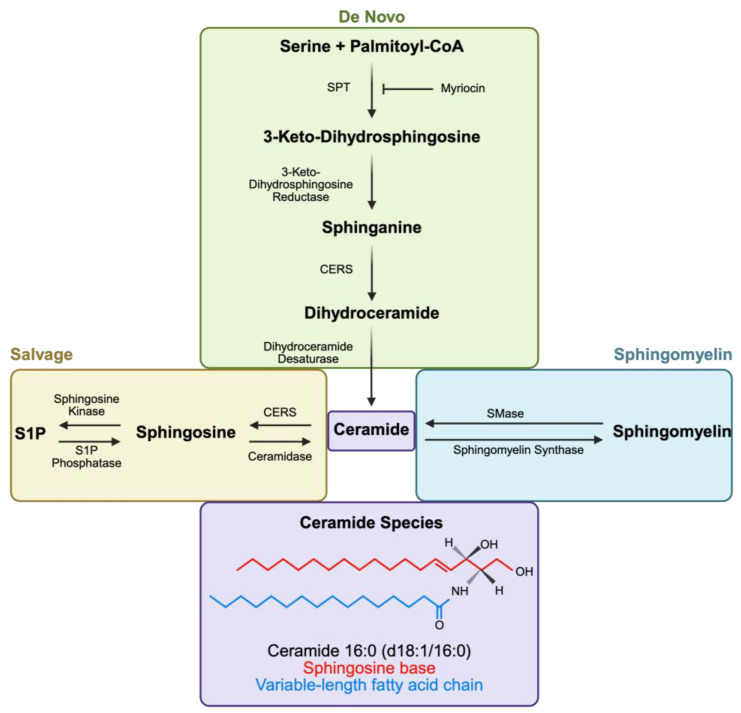
Pathways of ceramide synthesis and degradation. SPT: serine palmitoyl transferase; CERS: ceramide synthase; S1P: sphingosine-1-phosphate; SMase: sphingomyelinase. Created in BioRender. Available online: https://BioRender.com/ (accessed on 1 August 2025).

A ceramide molecule is composed of a sphingosine backbone and a fatty acid linked together by an amide bond [17]. The biochemical synthesis of ceramides within the cell has been extensively described in previous reports [18]. An overview of this process is depicted in Figure 1. In summary, ceramide production occurs through three pathways: de novo synthesis, the salvage pathway, and the sphingomyelinase pathway. The de novo pathway takes place on the cytosolic surface of the endoplasmic reticulum (ER) and utilizes palmitoyl-CoA and serine to form 3-keto-dihydrosphingosine via serine palmitoyltransferase (SPT) activity [19,20]. Next, 3-keto-dihydrosphingosine reductase generates sphinganine, which combines with one of six isoforms of ceramide synthase (CERS). Ceramide synthases are localized to different tissues, and each isoform preferentially utilizes fatty acyl-coenzyme A (CoA) of specific chain lengths to generate unique ceramide species [21]. Ultimately, CERS enzymes catalyze the n-acylation of sphinganine to produce dihydroceramide (DhCer) [22]. The final step of synthesis introduces a 4,5-trans-double bond—the unique functional aspect of ceramide—into the sphingoid backbone of the dihydroceramide [22]. The final product, ceramide, can then be translocated to the Golgi apparatus to produce more complex sphingolipids via specific ceramide transport proteins (CERTs) [23]. Alternatively, the salvage pathway functions by reacylating the sphingosine generated by the breakdown of complex sphingolipids. In the sphingomyelinase pathway, ceramides can be reproduced through the action of sphingomyelinases (SMases), removing the choline head group of sphingomyelin [18]. Catabolism of ceramide generates sphingosine via the work of ceramidases. Phosphorylation of sphingosine by sphingosine kinases can generate sphingosine-1-phosphate (S1P). S1P acts as both an extracellular and intracellular signal, influencing cell survival, proliferation, and immune regulation [24].

Long-chain ceramides (e.g., C16:0) have been associated with increased cardiovascular and metabolic disease risk, whereas very-long-chain ceramides (e.g., C22:0, C24:0) often correlate with protective outcomes in metabolic tissues [25,26,27,28,29,30]. On the contrary, very-long-chain ceramides like C22:0 and C24:0—as well as the ratio of C24:0 to C16:0 ceramide—are inversely correlated with cardiovascular risk [31,32,33]. These patterns have been key in developing clinical risk-prediction tools like CERT1, CERT2, the diabetes risk dScore, and the SIC score, which use a patient’s plasma ceramide profile to stratify risk of cardiometabolic disease [18,34,35,36,37]. Notably, interventions that alter ceramide species profiles (such as diet or exercise) could potentially modulate these risk scores, highlighting the clinical relevance of understanding ceramide species in MASLD.

### 2.2. Normal Mitochondrial Function

The mitochondrion is the main supplier of energy for cells. Acetyl-CoA is the common intermediate that enters the tricarboxylic acid (TCA) cycle to generate adenosine triphosphate (ATP). Acetyl-CoA may be generated from pyruvate during glucose catabolism or from fatty acids during β-oxidation. In hepatocytes, free fatty acids (FFAs) are first converted to fatty acyl-CoA in the cytosol and subsequently transported into the mitochondria via the carnitine shuttle. β-oxidation breaks down the fatty acyl-CoA into multiple acetyl-CoA molecules, which can then be further oxidized to CO_2_ and H_2_O in the TCA cycle, ultimately producing ATP.

The electron transport chain (ETC) is located within the inner mitochondrial membrane and serves as the primary site of ATP production through oxidative phosphorylation. The ETC is composed of five complexes, numbered I through V. The electron carriers NADH and FADH2 serve as reducing components for this aerobic process. By transferring electrons from complex I to complex V, energy is provided to pump protons from the mitochondrial matrix to the intermembranous space of the organelle, resulting in a proton gradient. The proton gradient is then used by complex V (ATP synthase) for the synthesis of ATP from adenosine diphosphate and inorganic phosphate. When protons leak from the intermembrane space, the mitochondrial transmembrane potential (Δ Ψm) decreases, and heat is produced instead of ATP—an important physiological function of the organelle. To achieve this uncoupling, the TCA cycle and ETC must be partially uncoupled, which results in the generation of reactive oxygen species (ROS). Ideally, ROS are neutralized by enzymes like superoxide dismutase or glutathione peroxidase. However, if neutralization does not occur, ROS can cause mitochondrial dysfunction through oxidative damage and destruction of membranes, DNA, and proteins essential for normal functioning.

Mitochondrial dynamics involve the processes of fusion and fission of mitochondria [38]. These two opposing processes help maintain organelle function during times of metabolic or environmental stress. Fusion occurs when the contents of partially damaged mitochondria are combined to promote complementation, thereby mitigating cellular stress. Mitofusin-1 (Mfn1) and mitofusin-2 (Mfn2) are responsible for fusion of the outer mitochondrial membrane (OMM) [39], while the optic atrophy 1 (Opa1) protein regulates the inner mitochondrial membrane (IMM) [40,41]. Fission is a complementary quality-control process that separates damaged mitochondria from their healthy counterparts, creating new mitochondria through division of pre-existing ones [42,43]. In fission, the primary proteins include dynamin-related protein 1 (Drp1) and mitochondrial fission 1 protein (Fis1) [44,45]. Disruptions in the balance of fusion and fission, or within the processes themselves, have been implicated in disease [46]. These disruptions can originate from intracellular stress, as well as external factors, resulting in mitochondrial fragmentation [47].

Mitophagy is mitochondria-specific autophagy involving the selective isolation and degradation of damaged mitochondria [48]. This process maintains the functional integrity and homeostasis of the cell and serves as a protective mechanism to avoid excess ROS generation from damaged mitochondrial contents [49]. In relation to metabolism, mitophagy can stimulate lipid droplet breakdown and release of FFAs, which are then shuttled to healthy mitochondria for energy production. The PTEN-induced kinase 1 (PINK1)–Parkin pathway is one of the most relevant mechanisms for mitophagy [50]. When mitochondrial membrane potential is lost, PINK1 accumulates on the OMM and recruits/autophosphorylates Parkin, activating the autophagic machinery.

Mitochondrial biogenesis is the process by which new mitochondria are formed within a cell. It requires transcription and replication of mitochondrial DNA (mtDNA), transcription of nuclear-encoded genes, and translation and assembly of the OXPHOS complex [51]. All these processes are essential to cellular homeostasis and survival. The main regulator of mitochondrial biogenesis is peroxisome proliferator-activated receptor gamma coactivator-1α (PGC-1α). In addition to being a major transcriptional coactivator, PGC-1α regulates mitochondrial biogenesis by modulating the expression of both nuclear and mitochondrial genes [52]. Through the manipulation of nuclear respiratory factors 1 and 2 (NRF-1 and NRF-2), expression of transcription factor A (TFAM) is increased, which directly interacts with the mitochondrial genome to initiate transcription and replication of mtDNA [53,54,55]. PGC-1α increases mitochondrial fatty acid oxidation (FAO) gene expression through co-activation of peroxisome proliferator-activated receptors α and γ (PPARα and PPARγ). Furthermore, PGC-1α stimulates mitochondrial biogenesis through coordinated upregulation of genes involved in mtDNA replication and gene expression [56,57]. PGC-1α and sirtuins (SIRTs), another family of effector enzymes in mitochondria consisting of NAD-dependent protein deacetylases, are able to regulate each other [58]. SIRT3, one of the most well-studied SIRTs, controls ATP synthesis through the regulation of complexes I and II, activates β-oxidation enzymes and acetyl-CoA synthetase, and prevents oxidative damage through interactions with superoxide dismutase 2 (SOD2) and isocitrate dehydrogenase 2 (IDH2) [59,60,61]. Both PGC-1α and SIRT3 are downstream of AMP-activated protein kinase (AMPK), a key regulator of fatty acid metabolism in the liver [62].

### 2.3. Localization of Ceramides in the Mitochondria

Ceramides were first discovered in mitochondrial membranes in the early 2000s and have since been shown to localize to both the OMM and IMM, with each membrane being associated with specific ceramide species [63,64]. Ceramides preferentially relocate to and accumulate in cells that are experimentally programmed for apoptosis [65]. Mitochondria are a main target in ceramide-mediated apoptosis: apoptotic stress signals activate both the sphingomyelin and de novo synthesis pathways of ceramide synthesis [66,67].

Ceramides in the mitochondrial membrane are hypothesized to originate from two main sources. The first and most common source involves the translocation of ceramides from the endoplasmic reticulum (ER) to the mitochondria. The ER has a specialized sub-compartment termed the mitochondria-associated membrane (MAM) that contains CERS enzymes and glycosyltransferases [68,69]. Ceramides generated in the MAM are transferred to the mitochondria via intimate contact between the two organelles [70,71]. The second source of mitochondrial ceramides is synthesis within the organelle itself. CERS, SMase, and reverse ceramidase activities have been demonstrated in the mitochondrial membranes [8,72]. Experimental data from isolated mitochondria show that MAMs are able to generate sufficient ceramide—via ceramide synthases or the salvage pathway—to disrupt the integrity of the OMM [73,74]. This supports the notion of localized ceramide synthesis in mitochondria, though this is likely less common than translocation from the ER. The presence of ceramide enzymatic activity within mitochondria indicates a unique pool of ceramide that may regulate mitochondrial apoptosis or mitophagy through direct contact [7,75,76,77].

### 2.4. The Role of Ceramides in Mitochondria

**Figure 2:**
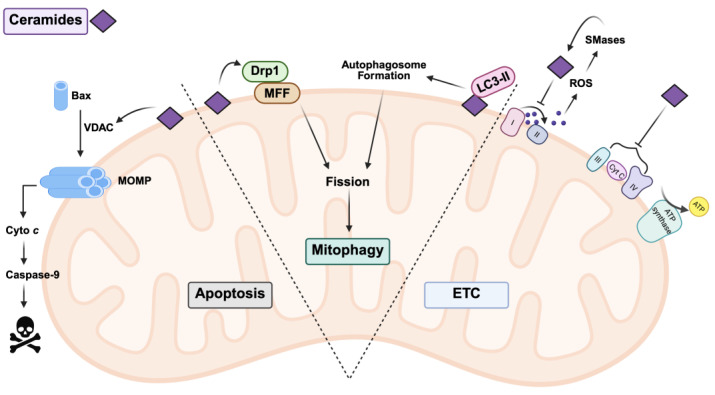
The role of ceramides in mitochondria. VDAC: voltage-dependent anion channel; MOMP: mitochondrial outer membrane permeabilization; Drp1: dynamin-related protein 1; MFF: mitochondrial fusion factor; LC3-II: microtubule-associated protein 1 light chain 3; ROS: reactive oxygen species (generated via electron (purple dots) leakage from the ETC); ETC: electron transport chain; SMases: sphingomyelinases; ATP: adenosine triphosphate. Created in BioRender. Available online: https://BioRender.com/ (accessed on 1 August 2025).

Several animal studies using myriocin, a potent SPT inhibitor, have shown that inhibiting sphingolipid synthesis can protect against cell death, supporting the idea that excessive ceramide accumulation may promote apoptosis [78]. However, other studies have revealed that proper ceramide metabolism can protect cells from apoptotic stimuli, including ceramide-induced apoptosis [79]. Together, these findings suggest that while ceramides may be necessary for normal mitochondrial processes, their dysregulation can lead to mitochondrial dysfunction and apoptosis. As shown in Figure 2, ceramides are well studied in the process of apoptosis but also have impacts on the ETC and mitophagy.

Apoptosis is a programmed cell death mechanism by which multicellular organisms remove damaged or unnecessary cells. This process is initiated by either the extrinsic pathway (using extracellular signals like Fas ligand and tumor necrosis factor, TNF) or the intrinsic pathway (triggered by DNA damage and oxidative stress) [80]. Mitochondria respond to intrinsic apoptotic signals by initiating mitochondrial outer membrane permeabilization (MOMP). This involves the release of pro-apoptotic factors and the activation of caspases into the cytoplasm, ultimately leading to cell death [80]. Ceramides have been shown to induce this process not just when added exogenously to cells but also when endogenous ceramide levels are increased [81]. The translocation of ceramides to mitochondria further promotes mitochondrial ceramide accumulation and apoptotic induction [65,82]. In response to increased ceramide levels, the permeability of the OMM increases in parallel [83]. Ceramide-induced apoptosis is associated with increased ROS, which, in turn, causes widespread mitochondrial dysfunction. In a cyclic nature, this dysfunction may cause a collapse in MOMP, furthering mitochondrial failure and activating the intrinsic apoptotic pathway [84]. It is important to note that ceramides themselves are not sufficient to initiate apoptosis; ceramides work synergistically with pro-apoptotic Bcl-2 family proteins (e.g., Bax). The Bax protein has been shown to utilize ceramide-generated pores (particularly those formed by C16:0 ceramide) to integrate into the OMM and induce the loss of the mitochondrial membrane potential necessary for cell death [85,86,87]. There is evidence that ceramides may promote the translocation of Bax to mitochondrial membranes [88]. Ceramides may also mediate pro-apoptotic activity through the voltage-dependent anion channel VDAC2, a mitochondrial platform for Bax translocation. It has been demonstrated that ceramides may bind to VDAC2 to exert their effects [89].

Ceramides play a significant role in determining mitochondrial fate and REDOX balance through effects on mitophagy and biogenesis [48,90,91,92]. Mitophagy can be grouped into protective and lethal processes. Protective mitophagy maintains appropriate energy levels through the clearance of dysfunctional mitochondria, whereas excessive (lethal) mitophagy is associated with complete cell death [93]. A current hypothesis implicates ceramides as inducers of lethal mitophagy, whereas PINK1 mediates protective mitophagy [93]. In PINK1-deficient mammalian models, the accumulation of mitochondrial ceramides was observed to initiate mitophagy [94,95]. Before mitophagy can occur, damaged mitochondria are segregated by fission proteins into healthy and damaged portions. Ceramide-induced mitophagy requires mitochondrial fission, likely due to ceramides’ interactions with Drp1. Drp1 is both an essential regulator of fission and a recruiter of ceramides to the OMM [96]. Evidence shows that inhibiting mitochondrial fission or upregulating fusion diminishes ceramide-induced mitophagy [97,98]. Ceramides have also been shown to regulate mitophagy by serving as receptors on mitochondrial membranes for LC3-II–containing autophagosomes [96]. In experiments where sphingosine kinase 1 was inhibited, ceramide accumulation was associated with Drp1 upregulation and degradation of PINK1 and Mfn2 [99]. This suggests that ceramide-mediated mitophagy is associated with increased mitochondrial fission, potentially regulated by Drp1.

Interestingly, C18:0 ceramide (produced by CERS1) has been shown to induce mitophagy independent of Bax or relevant caspases in human cancer cells [96]. Ceramides C18:0 and C16:0 have also been shown to act as receptors on mitochondrial membranes to recruit and bind mitophagy-related autophagosomes. Indeed, some reports have indicated that subcellular localization of ceramides may be more important than acyl chain length in determining their role in mitophagy [96]. Paradoxically, cells sometimes upregulate mitochondrial biogenesis in what appears to be a compensatory response to ceramide toxicity [100]. In cell models, for example, exposure to ceramides has been shown to enhance PGC-1α expression, seemingly to counteract mitochondrial dysfunction caused by excessive mitophagy and fission [93]. More insight into the mechanisms of this relationship is needed, but the implication of ceramides in mitophagy is clear.

Ceramides also play a regulatory role in mitochondrial respiration. Several in vivo studies support the idea that CERS enzymes and other sphingolipid mediators are involved in normal ETC function [28,101]. Dysregulation in this process may result in the generation of ROS, which have a bidirectional relationship with ceramides. On one side, ceramides cause oxidative stress through the activation of NADPH oxidase and nitric oxide synthase, which induces mitochondrial dysfunction and the suppression of antioxidant enzymes [102,103,104]. On the other side, ROS produced due to extracellular stressors causes the accumulation of ceramides via an enhancement in SMase activity [102]. Long-chain ceramides like C16:0 have been shown to disrupt ETC complexes, leading to electron leakage and further ROS production [26]. Increases in C16:0 (often via CERS6 activity) and decreases in very-long-chain species (via reduced CERS2 activity) caused mice to develop early hepatocyte apoptosis and steatohepatitis [15,26]. If left unchecked, this cycle can lead to significant oxidative stress, cell death, and even organ dysfunction. Ceramides have been shown to interact with all four complexes of the ETC, as well as ANT1, influencing electron flux and promoting ROS leakage [26,103].

## 3. The Role of Ceramides and Mitochondria in MASLD

**Figure 3:**
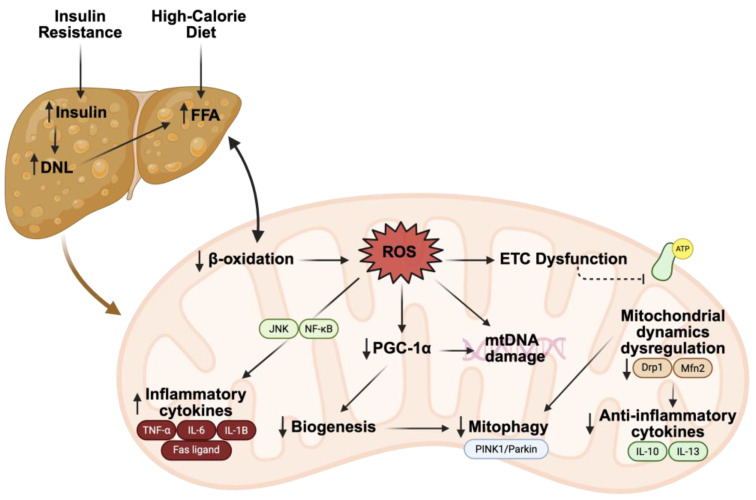
Mitochondrial dysfunction in the pathogenesis of MASLD. FFA: free fatty acid; DNL: de novo lipogenesis; JNK: c-jun N terminal kinase; NF-κB: nuclear factor-κB; IL: interleukin; TNF-α: tumor necrosis factor α; ROS: reactive oxygen species; ETC: electron transport chain; PINK: PTEN-induced kinase 1; Drp1: dynamin-related protein 1; Mfn2: mitofusin 2; mtDNA: mitochondrial DNA. Created in BioRender. Available online: https://BioRender.com/ (accessed on 1 August 2025).

The accumulation of ceramides within mitochondria has a similar effect to that of the mitochondrial dysfunction characteristic of MASLD. The decline in mitochondrial health contributes to lipid accumulation, inflammation, and potentially liver fibrosis. A summary of MASLD pathogenesis with an emphasis on the relationship between the liver and mitochondria may be found in Figure 3.

### 3.1. Pathogenesis of MASLD

The development of MASLD is currently understood as a culmination of multiple “hits”. Evolving from the earlier “two-hit” hypothesis, the current “multiple-hit” hypothesis considers genetic susceptibility, insulin resistance, gut microbiota, diet, and sedentary behavior as factors that can contribute to MASLD [105,106]. The relative contribution of these factors to MASLD pathogenesis varies by individual; however, excess caloric intake and the subsequent storage of triglycerides (TGs) and lipids as adipose tissue are hallmarks of MASLD pathogenesis [107]. In MASLD, the primary metabolic dysregulation is an increase in lipolysis and non-esterified fatty acids (FFAs) in the bloodstream. Patients with MASLD have elevated levels of FFAs, TGs, ceramides, and bile acids in the blood, while phospholipids are often depleted [108,109,110,111]. A variety of fatty acid sources contribute to hepatic lipid accumulation in MASLD. Approximately 59% of FFAs stored in the liver originate from adipose tissue lipolysis, 26% come from de novo lipogenesis (DNL), and 15% derive from dietary sources [112]. FFAs can be converted to TGs for storage or exported from the liver as very-low-density lipoproteins (VLDLs) [113]. Excessive and pathological hepatic lipid accumulation in MASLD has been attributed to at least three processes: increased visceral adipose tissue lipolysis, enhanced hepatic DNL, and high-calorie dietary intake [112].

Due to increased circulating FFAs in MASLD, the body often develops peripheral insulin resistance—a key contributor to MASLD progression [114,115]. Insulin resistance increases hepatic DNL, promotes secretion of inflammatory cytokines and adipokines, and exacerbates dysregulation of adipose tissue lipolysis [116]. As insulin resistance progresses, fat continues to accumulate in the liver, causing lipotoxicity—a condition that promotes oxidative stress and impairs mitochondrial and cellular function [117]. The accumulation of ROS can lead to peroxidative attack on mitochondrial membrane lipids (lipid peroxidation) [118,119]. These damages, in turn, promote apoptosis and necrosis as a means of protection. Hepatic stellate cells (HSCs) are activated in response to such damage and can promote fibrosis and cirrhosis via differentiation into collagen-producing myofibroblasts [120].

A continuous inter-organ crosstalk between adipose tissue and the gut may also contribute to MASLD development [121]. It is proposed that an imbalance in the gut microbiota as a result of caloric excess leads to increased bacterial products in the portal circulation. These microbial products (e.g., lipopolysaccharide) can activate the hepatic innate immune system and contribute to liver injury [122]. MASLD pathogenesis is also influenced by genetic and epigenetic factors, with heritability estimates ranging from 20% to 70% [123,124]. Genome-wide association studies have revealed multiple single-nucleotide polymorphisms in genes such as *PNPLA3* that are independently correlated with the development and severity of MASLD [125].

High carbohydrate consumption has been shown to increase hepatic DNL, which, in turn, increases FFA synthesis and uptake, ultimately leading to fatty liver disease [126]. In a study of MASLD patients, abnormally high levels of DNL were documented [127]. This aligns with the finding that sterol regulatory element-binding protein 1c (SREBP-1c), a transcription factor that regulates DNL, has increased expression in MASLD patients and is inducible by a high-carbohydrate diet [128,129]. As the body’s immediate energy needs are met, additional energy is stored, and excess FFAs are shunted into nonoxidative pathways like sphingolipid synthesis. Signaling molecules of this pathway, mainly ceramides and S1P, indicate an over-nourished status and help cells adapt to the oversupply of FFAs [18,130].

### 3.2. FAO and ROS in MASLD

Increased DNL and reduced β-oxidation contribute to the excess lipid characteristic of the liver in MASLD. Hepatic mitochondrial dysfunction has been shown to precede the onset of MASLD in obese rodent models [131,132]. In obese patients with MASLD, the initial rise in hepatic lipid content can enhance mitochondrial activity; however, chronic lipid overload eventually leads to elevated oxidative stress and diminished organelle function [133].

Prior reports demonstrate that defects in mitochondrial FAO can induce hepatic steatosis in mice without progression to cirrhosis [134,135]. In humans, one study used liver biopsies from obese patients undergoing bariatric surgery to correlate liver histology with mitochondrial FAO measured in liver tissue samples. The study showed that hepatic mitochondrial complete FAO was reduced by ~40–50% in subjects with MASH compared to controls with normal liver histology [136]. The decline in FAO was accompanied by increased hepatic mitochondrial ROS production, as well as markers of mitochondrial biogenesis and mitophagy. These findings reinforce the relationship between mitochondrial dysfunction and MASLD in humans and suggest that impaired hepatic FAO and reduced mitochondrial health are strongly linked to increased MASLD severity in patients with obesity.

In the early stages of disease, mitochondrial dysfunction activates multiple pathways to increase mitochondrial activity and reduce oxidative damage. This compensatory upregulation is mediated through increased expression of ROS-detoxification genes via upregulation of PGC-1α, SIRT1, and SIRT3 [137]. As the disease progresses, hepatic mitochondrial depletion and dysfunction occur. When mitochondrial fatty acid oxidation is impaired, alternative peroxisomal and cytochrome P450 oxidation of FFAs ensues [138,139]. This results in significant ROS production and other toxic byproducts. Excess ROS in MASLD is associated with ETC disruption, MOMP induction, altered mitochondrial membrane potential, and structural mitochondrial abnormalities [134,135]. More specifically, individuals with MASLD were found to have reductions in respiratory chain activity of 37% in complex I, 42% in complex II, 30% in complex III, 38% in complex IV, and 58% in complex V [140]. ROS also causes oxidative base lesions in mtDNA, which lead to further ROS leakage due to direct damage to electron transport integrity [141,142,143]. Patients with MASLD tend to have a higher rate of mtDNA mutations affecting ETC complexes, with mutational burden worsening in parallel with disease severity [144,145]. The initiation of this harmful ROS-mediated cycle of damage can be partly attributed to long-chain FFAs that accumulate in hepatocytes in MASLD. In a rodent model of MASLD, ultrastructural impacts associated with excess ROS production included disrupted cristae, hypodense matrix, and swollen/rounded mitochondria [138,139]. Similarly, in humans with MASLD, ultrastructurally abnormal mitochondria have been observed in liver tissue, alongside higher levels of ROS and ROS-mediated mtDNA damage [133,141,146,147].

Increased FFA levels due to MASLD can also activate inflammatory pathways within hepatocytes, leading to liver injury [148]. Increased ROS in the hepatocyte directly induces inflammation. This begins with upregulation of gene expression for cytokines like TNF-α and Fas ligand, which enact their effects through inflammatory signaling pathways like NF-κB and JNK [149]. In addition to NF-κB, ROS also stimulate the nuclear-binding oligomerization domain-like receptor family and the pyrin domain containing 3 (NLRP3) inflammasome, producing inflammatory cytokines such as IL-1B, IL-6, and TNF-α [150]. The release of cytochrome C can be upregulated by TNF-α, which, in turn, causes increased apoptosis and necrosis [151]. The mitochondrial membrane disruption induced by ROS facilitates the creation of MPTPs, which release mtDNA into the cytoplasm and further hepatocyte necrosis and inflammation [152,153,154].

### 3.3. Biogenesis, Mitophagy, and Dynamics in MASLD

Biogenesis, mitophagy, and mitochondrial dynamics are active components of the mitochondrion’s drive to maintain cellular homeostasis and control the quality of its products. To regulate these processes, AMPK uses SIRT3 and PGC-1α as downstream effectors, and these two mediators are able to regulate each other [58]. In MASLD, hepatocytes have been shown to have decreased AMPK activity, resulting in mitochondrial dysfunction [155]. In addition, the mRNA expression of PGC-1α is decreased in MASLD patients, with a subsequent decrease in hepatic mitochondrial respiration [133]. SIRTs are also downregulated in MASLD patients, leading to hyperacetylation of various mitochondrial proteins and disruption of their function [156,157]. Overexpression of SIRT3 in cell culture experiments has been shown to enhance mitochondrial respiratory capacity and redox homeostasis, reducing hepatic lipid accumulation and oxidative stress [158]. In a rodent model of in vivo SIRT3 overexpression (in the setting of heterozygosity for a β-oxidation defect), a significant increase in mitochondrial FAO was observed, which prevented MASLD in the otherwise susceptible mice [159].

Mitochondrial fission, mediated by Drp1, can be induced by a high-fat diet. Excessive fission is associated with lower levels of anti-inflammatory cytokines, like interleukin-10 (IL-10) and IL-13, implicating mitochondrial fission in the development of hepatic inflammation [104]. In a rodent model of MASH induced by long-term Western diet feeding, an observable decrease in Fis1 and Drp1 protein levels was seen, resulting in hepatic inflammation and fibrosis [160]. In biopsy samples of MASH patients, Mfn2 levels tend to be decreased, which aligns with findings in animal models [161]. Animal models have also shown that Mfn2 reexpression reduces liver disease, while deletion of Mfn2 causes inflammation, TG accumulation, fibrosis, and liver cancer [161].

The accumulation of damaged and defective mitochondria, a state typical of advanced MASLD, may be due, in part, to alterations in mitophagy. Liver mitochondria of Bnip3-deficient mice, which have impaired mitophagy, exhibited decreased Δ Ψm, abnormal morphology, and reduced oxygen consumption [162]. These defects were associated with increased ROS, inflammation, and steatohepatitis-like histological abnormalities. Additionally, a high-fat diet (HFD)-fed MASLD rodent model showed downregulation of PINK1 and Parkin, with associated activation of the apoptotic pathway and mPTP opening [163].

As mentioned, mitochondrial dysfunction in the early stages of MASLD triggers adaptive pathways to reduce oxidative damage, including upregulation of antioxidant defenses. As disease severity progresses, mitophagy is eventually increased while mtDNA and PGC-1α expression are decreased. This contributes to a destructive cycle of hepatic mitochondrial damage and dysfunction. Inappropriate regulation of mitophagy promotes further oxidative stress and inflammation, which, in turn, furthers the inability to adequately clear or replace damaged mitochondria.

#### 3.3.1. Ceramide Concentrations in MASLD

In animal models of MASLD, an increase in total liver ceramide concentrations is observed in most, but not all, studies [164,165,166]. One explanation for inconsistent findings is that hepatic intracellular ceramide levels are tightly regulated. To prevent toxic intra-organ buildup, the liver may increase ceramide secretion into the circulation via VLDL lipoproteins as a protective mechanism [167]. A second explanation for the lack of total liver ceramide increase in some MASLD models is the possible formation of an “intracellular inactive ceramide storage pool”. A recent study showed that hepatic lipotoxicity enhanced the conversion of biologically active ceramides to inactive acylceramides, creating a storage pool that prevents ceramides from accumulating in liver membranes [168,169].

A notable trend is observed regarding ceramide species in MASLD. Steatosis is associated with increases in ceramides C14:0, C16:0, C18:0, and C20:0, along with a decrease in very-long-chain species like C24:0. These trends have been linked to a parallel increase in the de novo synthesis pathway and expression of SPT and CERS1, 2, 4, and 6 [12,14,15,170]. However, changes in CERS activity do not always correlate exactly with their associated ceramide species [31]. Acid SMase activity (salvage pathway) has also been related to increased ceramide concentrations in MASLD [171].

Data on liver ceramide concentrations in humans are comparatively limited. One study compared the lipid content of liver and plasma samples from patients grouped as healthy, simple steatosis, NASH, or cirrhosis [172]. Differences in liver ceramide concentrations were negligible between the four groups. In plasma, the levels of ceramide C18:0, 20:0, 22:0, 24:0, 24:1, and DhCer C18:0 and 24:1 were comparable between the steatotic and healthy subjects, increased in the NASH subjects, and lower in the cirrhotic group. A separate study found an increase in hepatic total ceramide and DhCer (dihydroceramide) C16:0, 22:0, and 24:1 in individuals with NASH, regardless of whether they were categorized as lean or insulin-resistant obese [173]. Another study, which focused on liver samples of patients undergoing bariatric surgery, stratified data based on the degree of insulin resistance (HOMA-IR) and liver fat content. Ceramides C16:0, 18:0, 22:0, and 24:1 were increased in the livers of patients with both high insulin resistance and high liver fat. Also increased were hepatic palmitate concentrations and markers of de novo synthesis for DhCer C16:0, 18:0, 23:0, and 24:1. Markers of the salvage pathway and sphingomyelin hydrolysis were not significantly increased, pointing to the de novo pathway as primarily responsible for the increase in hepatic ceramides [174]. In a more recent sample of 73 MASLD patients, the expression of sphingolipid de novo synthesis enzymes was found to increase concomitantly with disease severity [175]. Overall, hepatic ceramide concentrations appear to be elevated in humans with MASLD, with further increases in the more advanced state of MASH.

#### 3.3.2. Ceramides and Insulin Resistance in MASLD

Ceramides have been implicated in the development of insulin resistance, a major driver of hepatic steatosis. This relationship in skeletal muscle and adipose tissue has been well elucidated [176,177]. In the liver, ceramides’ main target in the insulin signaling pathway is the inhibition of Akt. Normally, Akt acts to promote glucose uptake and inhibit gluconeogenesis. Ceramides activate PKCζ, which phosphorylates Akt on a Thr34/Ser34 residue, inducing sequestration of Akt into caveolae—specialized invaginations of the plasma membrane. Once sequestered in caveolae, Akt’s ability to activate in response to insulin is impaired [178,179,180,181]. Ceramides may also inhibit Akt by a second mechanism: ceramide-activated protein phosphatase 2A (PP2A) can dephosphorylate Akt at critical activation sites, as shown in cells lacking caveolae [179,182,183]. The full effects of ceramides on hepatic insulin signaling have yet to be completely revealed. It is hypothesized that, due to the low density of caveolae in hepatocytes, the PP2A axis may predominate. By inducing hepatic insulin resistance, ceramides promote a state of elevated gluconeogenesis and hyperinsulinemia, which directly drives de novo lipid synthesis in the liver [169,174].

Regarding the liver, a rodent model of CERS2 (very-long-chain ceramide synthase) haploinsufficiency showed a decrease in very-long-chain species (C22:0, C24:0, and 24:1), with a compensatory increase in C16:0 ceramides, leading to HFD-induced insulin resistance [184]. Separately, CERS2-null mice exhibited glucose intolerance and dysfunctional insulin receptors in the liver but not in skeletal muscle or adipose tissue [185]. Conversely, overexpression of CERS2 in mice improved hepatic insulin signaling [32]. Further studies have implicated CERS6 and its C16:0 ceramides in insulin resistance [15,26]. In models of CERS6 overexpression, hepatic Akt phosphorylation was inhibited and TG accumulation was promoted [15]. Desaturase-1 (DES1) is an enzyme that produces ceramides from DhCER. A liver-specific deletion of DES1 prevented insulin resistance in both leptin-deficient and HFD-fed mice, further implicating ceramides in glucose homeostasis [27]. These data support C16:0 ceramides as deleterious species, whereas very-long-chain ceramides (C22:0–C24:0) have more protective effects. Modulating the balance of these species and their associated enzymes could be a viable strategy to improve insulin sensitivity in MASLD patients [186].

#### 3.3.3. Ceramides That Increase Fatty Acid Availability

Ceramides may contribute in several ways to the lipid overload characteristic of hepatocytes in MASLD. These mechanisms include increased fatty acid uptake, increased lipogenesis, and decreased fatty acid oxidation.

As previously mentioned, lipogenesis and fatty acid utilization are dependent on the nutritional energy status of the body. Individuals with MASLD are often in a state of nutrient excess. As a result, plasma FFAs—especially saturated fatty acids—are elevated in individuals with MASLD and MASH [127,187]. In humans with MASLD, around 60% of hepatic TGs are synthesized from plasma FFAs [112]. Hepatocyte uptake of FFAs is mediated by CD36 (also known as fatty acid translocase) and a family of fatty acid transport proteins (FATPs), with FATP2 and FATP5 being the most active in the liver [188]. These transporters act in conjunction with acyl-CoA synthetase to import fatty acyl-CoA into cells. In obese rodent models, artificially lowering hepatic ceramide concentrations (through overexpression of ceramidase) led to decreased fatty acid uptake [18,189]. In the absence of ceramide-induced PKCζ activation, there was a subsequent decrease in expression of CD36, FATP2, and FATP5, as well as reduced translocation of CD36 to the plasma membrane [189]. In a model of acid ceramidase overexpression, hepatic C16:0 and C18:0 ceramides were reduced along with CD36 expression, supporting the idea that ceramides promote fatty acid uptake in the liver [189].

Lipogenesis (de novo fatty acid synthesis from carbohydrate) is regulated by both energy status and transcriptional control by glucose and insulin. SREBP-1c is a key transcription factor activated by insulin, and ChREBP is activated by glucose. In both animal models [190,191] and humans [192,193,194] with MASLD, the expression of SREBP-1c and its target genes is elevated. Regarding ceramides, the expression of certain CERS enzymes (notably CERS6) is upregulated by a high-carbohydrate diet, linking excess carbohydrate intake to increased ceramide synthesis [195]. Ceramides themselves can further enhance lipogenesis: one study showed that ceramide infusion activated protein phosphatase PP2A, which stabilized the mature form of SREBP-1c, thereby increasing lipogenic gene expression [196]. Thus, ceramides can perpetuate a cycle of FFA oversupply and hepatic fat accumulation by both increasing FFA uptake and potentiating DNL.

In parallel, ceramides may impair fatty acid oxidation (FAO) in the liver. As described earlier, mitochondrial uptake and β-oxidation of FFAs are critical for preventing lipid accumulation. Ceramides have been shown to inhibit mitochondrial FAO. For example, C16:0 ceramide can directly interfere with the electron transport chain, reducing the efficiency of β-oxidation and leading to incomplete fatty acid oxidation [26]. Additionally, ceramide-mediated insulin resistance can indirectly reduce mitochondrial FAO by limiting insulin’s suppression of adipose lipolysis, thus flooding the liver with FFAs and overwhelming its oxidative capacity. By simultaneously increasing lipid input and impairing lipid utilization, ceramides create a scenario in which excess fatty acids are stored as hepatic TGs, exacerbating steatosis.

#### 3.3.4. Ceramides and MASH

Ceramides have additional effects that may contribute to the progression of MASLD to MASH through worsening inflammation, cell death, and fibrosis. The presence of MASH is characterized histologically by steatosis with superimposed inflammation and hepatocellular injury (ballooning), often accompanied by fibrosis. Ceramides, as pro-apoptotic and pro-inflammatory mediators, could influence each of these pathological features.

Tumor necrosis factor-alpha (TNF-α) plays a significant role in MASLD/MASH pathogenesis and has complex interactions with ceramide metabolism. TNF-α can activate SMases, leading to increased ceramide production, which, in turn, can amplify TNF-α signaling and hepatocyte apoptosis. In one study, mice lacking TNF-α signaling were protected from ceramide accumulation and liver injury on a high-fat diet, suggesting an intersection between cytokine and ceramide pathways in driving steatohepatitis [197]. Conversely, ceramide accumulation can stimulate the production of TNF-α and other cytokines via activation of NF-κB and other stress kinases, thereby sustaining a chronic inflammatory state in the liver. This feed-forward loop may underlie the transition from simple steatosis (largely benign) to steatohepatitis (inflammatory and progressive).

Fibrosis is a defense mechanism triggered by chronic liver injury, wherein activated HSCs deposit extracellular matrix to encapsulate damage. Ceramides have been implicated in fibrogenesis as well. Pharmacological or genetic inhibition of ceramide synthesis (for example, using myriocin to inhibit SPT) has been shown to improve hepatic fibrosis and inflammation in MASLD models [18]. However, the literature on ceramides in hepatic fibrosis is somewhat conflicting: different ceramide species and subcellular distributions may have divergent effects on HSC activation [169,198,199,200]. Some studies indicate that very-long-chain ceramides might play a protective role in HSCs, whereas C16:0 ceramide promotes a pro-fibrogenic phenotype [198]. Regardless, the cumulative impact of ceramides on apoptosis (driving hepatocyte loss), inflammation (recruiting immune cells), and HSC activation (laying down fibrotic tissue) likely contributes to the progression of MASLD toward cirrhosis.

In summary, ceramides contribute to MASLD pathophysiology on multiple fronts: metabolic (insulin resistance, altered lipid flux), oxidative (mitochondrial dysfunction, ROS generation), inflammatory (cytokine activation), and fibrogenic (HSC modulation). These effects place ceramide metabolism as a potential therapeutic target for preventing the escalation of steatosis to steatohepatitis and fibrosis in MASLD.

## 4. Diet Interventions in MASLD

Current knowledge on lifestyle interventions in MASLD is largely gathered from animal-based studies, and further research is needed to elucidate the exact effects in human patients. Below, we outline how various dietary strategies and exercise regimens may modulate ceramide synthesis and mitochondrial health in the context of MASLD management.

### 4.1. Clinical Benefits of Dietary Treatment in MASLD

The Western diet is a main driver of rising obesity rates worldwide [201]. This dietary pattern is characterized by high consumption of saturated fats, red and processed meats, refined grains, sweetened beverages, and a paucity of fruits, vegetables, and whole grains. The intake of fructose-containing drinks has been associated with a higher risk of MASLD with significant fibrosis, independent of total caloric intake [202]. In the chronic state of overnutrition typical of MASLD, fructose and other highly absorbable sugars are readily utilized in hepatic DNL, which worsens lipid accumulation and oxidative stress [129,203]. Modifying or limiting a Western diet pattern plays an important role in managing patients with MASLD.

The effects of weight loss on MASLD progression are well documented. In patients with fatty liver, a weight loss of 7% led to MASH resolution in close to 90% of patients, while a weight loss of ≥10% led to regression of fibrosis in ~45% of patients [204]. Any type of reasonable dietary pattern with both appropriate caloric restriction and long-term sustainability will improve the features of MASLD, as weight loss is the main physiological driver of benefit. Current clinical guidelines recommend weight loss via the Mediterranean diet (MD) for MASLD patients [205].

### 4.2. Energy Restriction and Macronutrient Composition

An energy-restricted diet, also referred to as a caloric restriction diet, has been well studied in the treatment of MASLD. This approach directly decreases the load of excess FFAs delivered to the liver. Energy restriction also enhances insulin sensitivity and lowers the incidence of diabetes and other metabolic disorders [206,207]. The clinical trial by Vilar-Gómez et al., involving 293 subjects with histologically proven MASH, provides one of the strongest pieces of evidence for using this strategy as a treatment for liver disease [204]. Subjects’ daily caloric intake was reduced by 750 kcal from baseline and comprised approximately 64% carbohydrates, 22% fats, and 14% protein. In addition, subjects were encouraged to increase their daily walking to 200 min per week. After one year of intervention, 25% of patients achieved complete MASH resolution with no worsening of fibrosis. The study’s results also suggest that sustained weight loss of >5% may reduce liver fat, but at least 7–10% weight loss is required to improve liver inflammation and >10% to improve fibrosis [204].

Our understanding of the relationship between caloric restriction, mitochondria, and ceramide is limited, consisting largely of pre-clinical studies in skeletal muscle and rodent models. Caloric restriction has been shown to decrease ceramide content in the livers of rodents, an effect enhanced by additional dietary protein and calcium supplementation [208]. However, this effect is likely tissue-specific, as no impact on myocardial ceramide levels was observed [209]. In a 16-week trial of caloric restriction, human subjects demonstrated decreased skeletal muscle ceramide levels [206]. Energy restriction has also been reported to reduce ROS formation, which may, in turn, reduce ceramide production [210]. In caloric restriction, the decreased intake of ceramide substrates (mainly saturated fatty acids) also likely contributes to the reduced de novo ceramide synthesis observed as a result of this regimen.

In a study of young non-obese adults, caloric restriction was associated with an increase in muscle mitochondrial DNA as well as a decrease in DNA damage. These improvements in mitochondrial health—in addition to enhanced autophagy—stem from the promotion of mitochondrial biogenesis. Caloric restriction enhances mitochondrial biogenesis and activates antioxidant defenses, such as Sirt3-dependent superoxide dismutase 2 (SOD2) [211,212,213]. The decrease in ceramides resulting from caloric restriction limits their usual pathogenic interactions, including inhibition of complex III (leading to ROS production), disruption of mitochondrial dynamics, and impairment of fatty acid oxidation.

Basic caloric restriction has been shown to be effective in MASLD resolution. However, intentional restriction of specific macronutrients may also be considered. The contrast between a carbohydrate-restricted diet and a fat-restricted diet is the most widely studied comparison in MASLD [214]. In a randomized clinical trial comparing these two strategies, similar weight loss was achieved in both groups, but a more significant reduction in liver fat occurred in the carbohydrate-restricted group [214]. In a separate study, the carbohydrate-restricted diet also led to lower ALT concentrations [215]. As previously mentioned, in one of the more extensive studies in this field, a 52-week trial implemented a low-fat diet plus moderate-intensity exercise and observed a decrease in steatohepatitis in participants who lost 7–10% of their weight [204]. There is substantial clinical support for both low-carbohydrate and low-fat diets in MASLD treatment, as both focus on hypocaloric implementation to drive weight loss and metabolic improvement [216]. In a fat-restricted diet, ceramide substrates (saturated fats) may be limited to a higher degree than in a carbohydrate-restricted diet. However, a conclusion on the differences in these diets and their potential effects on ceramide concentrations and clinical benefits in MASLD patients requires further study.

The main goal of caloric restriction is weight reduction, which then facilitates MASLD resolution. Individuals who undertake this dietary change may see short-term reductions in body weight and fat mass, yet most clinical studies show at least partial weight regain within 1–2 years [217]. Importantly, initial weight loss can have lasting benefits: a sustained improvement in hepatic fat content, liver function tests, and insulin resistance was observed in obese individuals two years after completing a 6-month hypocaloric diet, despite some weight regain [217].

### 4.3. Mediterranean Diet

The Mediterranean diet (MD) is the primary clinical recommendation of EASL–EASD–EASO guidelines for non-pharmacological intervention in MASLD [205]. The MD is favored over other strategies as it combines features of several regimens that have each been associated with improved MASLD outcomes. Its key features include minimally processed foods, low sugar content, and low saturated fat intake [205]. Adhering to the MD reduces saturated fat and animal protein consumption while increasing the intake of fiber, antioxidants, monounsaturated fatty acids (MUFAs), and polyphenols. Foods like extra-virgin olive oil, legumes, nuts, fruits, vegetables, fish, and moderate wine (with meals) are staples of the MD. Studies investigating the effect of the MD in MASLD patients have revealed significant improvements in disease markers, even in the absence of weight loss [218,219].

The effects of the MD on mitochondria are attributed to its bioactive compounds. These compounds improve overall mitochondrial health through modulation of oxidative phosphorylation, biogenesis, and mitophagy. In pre-clinical studies, pharmacological therapies for MASLD mimicked the effects of the MD’s bioactive compounds by promoting mitochondrial biogenesis and reducing oxidative damage [220,221,222].

Polyphenols, which are secondary plant metabolites, constitute a major class of bioactive components in the MD. Polyphenols are commonly classified into flavonoids and non-flavonoids based on chemical structure [138]. Through mitochondria-specific interactions, polyphenols can enhance ATP production, oxidative phosphorylation, and mitochondrial membrane stability in cell culture models. Furthermore, they contribute to reduced oxidative stress, as exhibited in multiple rodent models. In both cell culture and rodent models, polyphenols have been shown to enhance autophagy and mitophagy [223,224].

Hydroxytyrosol, a potent antioxidant found in olive oil, has been shown to reduce mitochondrial fission via downregulation of Drp1 and PPARγ while simultaneously promoting mitochondrial health in a model of obese mice [225]. Ferulic acid, found in a variety of fruits and vegetables, has been implicated in regulating mitochondrial dynamics through modulation of Mfn1, Mfn2, and Fis1 in rodent models [226]. The short-chain fatty acid butyrate, a product of dietary fiber fermentation, is present in significant amounts in the MD [227]. Its effects on mitochondrial health have been studied in insulin-resistant obese mice and include improving mitochondrial fusion, suppressing fission to maintain organelle integrity, and preventing mitochondrial dysfunction during metabolic stress. These actions occur through enhanced Mfn1, Mfn2, and Opa1, as well as reduced expression of Drp1 [227].

The PREDIMED trial (*Prevención con Dieta Mediterránea*) evaluated long-term effects of the MD compared to a control diet in a large cohort [228]. In an analysis of 980 participants from this trial, a “ceramide score” (a weighted sum of four plasma ceramide concentrations) was calculated and found to be associated with a 2.18-fold increased risk of cardiovascular disease (CVD). Notably, within the MD intervention group, individuals with high ceramide scores did not have higher CVD incidence compared to those with low ceramide scores. In the control diet group, however, a higher ceramide score conferred significantly increased CVD risk. This finding supports the hypothesis that the MD may beneficially influence disease-implicated ceramides and mitigate risk [229].

A recent randomized clinical study found that greater adherence to the MD was associated with a significant reduction in plasma ceramide concentrations at 6- and 12-month follow-ups. In addition, an increase in the C24:0/C16:0 ceramide ratio was observed—a metric typically associated with protective effects against cardiovascular disease [230]. Though the research on this topic is limited, there are several ways the MD may beneficially modulate ceramides. In cell culture and animal models, the presence of saturated FFAs promotes ceramide formation [231,232]. Conversely, exposure to unsaturated FFAs can prevent excess ceramide accumulation that is otherwise stimulated by saturated FFAs [233]. These findings are important, given that the Western diet is mainly composed of saturated fats (known stimulators of SPT activity). The MD’s high content of unsaturated fats may lessen this effect and thus limit de novo ceramide synthesis.

The stark difference in fatty acid composition between the Western diet and the MD is reflected in the FFA pool available for ceramide synthesis, potentially altering ceramide production. In a study on increased walnut consumption in humans, higher plasma sphingosine (a marker of ceramide degradation) was observed with no changes in dihydroceramide concentrations (a marker of ceramide synthesis). Walnuts are a key component of the MD, and the overall reduction in ceramides associated with walnut consumption could be due to increased ceramide catabolism, likely in addition to limiting substrate availability through decreased intake of saturated fatty acids [234]. The MD may also mitigate deleterious effects associated with ceramide accumulation through several mechanisms: directly lowering oxidative stress [235,236], reducing low-grade inflammation, and decreasing levels of oxidized LDL [237,238,239,240,241].

Greater adherence to the MD is associated with decreased insulin resistance and MASLD severity, as well as improved liver enzymes [242]. Epidemiological studies also indicate that the MD is associated with a lower likelihood of metabolic disease progressing to steatohepatitis [243]. Further clinical support comes from a study of obese diabetic patients placed on an MD: after nutritional counseling and implementation of the diet, the prevalence of hepatic steatosis in the cohort fell from 93% to 48%. A > 7% weight loss was achieved by 54.3% of participants, and the proportion of the cohort with abnormal liver enzymes dropped from 67% to 11% [244]. In an 18-month trial comparing the MD to a low-fat diet in obese patients, the MD produced greater decreases in hepatic fat content [245].

Overall, the Mediterranean diet serves as a beneficial dietary model for individuals with MASLD. Its reduced content of saturated fatty acids, richness in unsaturated fatty acids, and unique components (polyphenols, short-chain fatty acids, etc.) likely contribute to the observed improvements in liver steatosis, mitochondrial health, and metabolic parameters in MASLD patients.

### 4.4. Unique Dietary Components and Strategies

The ketogenic diet is another dietary strategy that could modulate ceramides to treat MASLD. The “keto” diet is a low-carbohydrate, high-fat diet that decreases appetite, facilitates weight loss, and helps regulate blood sugar levels in patients with insulin resistance. These effects are achieved mainly through diet-induced reductions in ghrelin and insulin secretion [246,247]. Across studies, implementation of the ketogenic diet in MASLD patients has shown beneficial weight loss and intrahepatic fat reduction. However, concerns have been raised about increases in LDL cholesterol and inflammatory markers in some patients [248,249,250]. In a recent pilot randomized controlled trial testing the effects of a ketogenic diet in MASLD patients, there was improvement in cardiovascular health markers without worsening of liver disease [251]. Still, the results and long-term benefits of this strategy with regard to potential adverse outcomes remain inconclusive. In a rodent model of ketogenic diet feeding, CERS6 was found to be downregulated while CERS2 was upregulated. Thus, the accumulation of long-chain ceramides, like C16:0, was minimized, and more beneficial very-long-chain ceramides in the liver were increased [252]. Additionally, PGC-1α was enhanced and TNF-α was reduced, consistent with observed reductions in lipogenesis and an overall anti-inflammatory effect. The ketogenic diet has also been shown to increase mitochondrial biogenesis, reduce inflammation, and boost fatty acid catabolism [253,254,255]. In turn, decreased substrate availability, increased lipid degradation capacity, and attenuation of inflammatory signaling would likely limit ceramide accumulation, though additional studies in human patients would be helpful in determining the exact effects.

Coffee consumption may also be beneficial for the treatment of MASLD. Increased coffee intake has been associated with a reduced risk of developing metabolic syndrome, liver fibrosis, type 2 diabetes mellitus, and cardiovascular disease [256,257,258]. An intake of ≥3 cups of coffee per day has been observed to correlate with a lower risk of MASLD compared to the consumption of <2 cups per day [259]. Further supporting this, a recent meta-analysis showed an association between regular coffee consumption and reduced hepatic fibrosis. One mechanism by which coffee may exert hepatoprotection is through inhibition of the mTOR complex, a key regulator of autophagy. Coffee, independent of caffeine, has been shown to inhibit mTOR in rodent models of diet-induced MASLD [260]. The effects of coffee on ceramide metabolism may underlie its beneficial impact on cardiometabolic and liver disease risk. Current evidence on this subject in humans revolves mainly around the modulation of specific ceramide species. Beneficial plasma ceramide species were found to be increased in an Asian population reporting high coffee consumption compared to a control group [261]. Specifically, coffee consumption was associated with increases in very-long-chain C24:0 ceramides in plasma. Another study showed coffee consumption associated with lower concentrations of Cer (d18:0/22:2) dihydroceramide—a species linked to increased cardiovascular disease risk [262]. In that analysis, adjusting for plasma dhCer22:2 attenuated the inverse association between coffee intake and type II diabetes risk by ~43%, supporting the idea that ceramide metabolism may mediate some dietary effects on disease risk. Modulation of lipids upstream from ceramides (for instance, via chlorogenic acid, a major coffee polyphenol) may contribute to decreased disease risk attributed to coffee. In animal studies, coffee has been implicated in beneficial lipid metabolic changes, with effects on SREBP-1, CD36, PPARα, and PPARγ [263,264,265,266]. The hepatoprotective effect of coffee has also been attributed to its polyphenol content, mainly chlorogenic acid. This compound has been shown to inhibit hepatic stellate cell activation in vitro; however, in a pilot study combining chlorogenic acid with caffeine, no significant effect on liver disease was observed in patients with type 2 diabetes [267,268]. Thus, while coffee contains components that can favorably influence pathways relevant to MASLD (ceramide metabolism, autophagy, inflammation), its exact benefits on liver outcomes may depend on complex interactions and require further investigation in clinical settings.

## 5. Exercise

Exercise-related changes significantly impact liver mitochondria in addition to skeletal muscle. Physical activity may indirectly modulate liver mitochondrial function, structure, and bioenergetics. The mechanisms of these effects include enhanced fatty acid β-oxidation, induction of autophagy, and upregulation of PPARγ signaling pathways [269,270].

### 5.1. Clinical Benefits of Exercise in MASLD

Increased physical activity to facilitate weight loss is critical in MASLD treatment [271]. Regardless of weight loss, exercise remains a first-line therapy for MASLD due to its direct beneficial effects on the liver and overall cardiometabolic health. Current recommendations for MASLD patients are to perform >150 min per week of moderate-intensity exercise or >75 min of vigorous-intensity exercise [205]. To be effective, exercise routines should be personalized to each patient to maximize adherence [272]. In a study of Chinese males, increased sedentary time was positively associated with MASLD prevalence [273]. Using both subjective (questionnaire) and objective (motion sensor) measures, MASLD patients were found to spend more time per day in sedentary activities than healthy controls, with BMI and body weight positively associated with sedentary behavior. Patients with MASLD were also shown to have fewer daily steps, lower total daily energy expenditure, and lower metabolic equivalent (MET) activity levels compared to healthy controls [274,275,276,277]. Insulin resistance and dysregulation of glucose homeostasis are major contributors to MASLD development, as established earlier. Physical activity can directly modulate these dysfunctions. Aerobic and resistance exercise programs, with or without concurrent weight loss, enhance peripheral insulin sensitivity. Skeletal muscle is a primary site for glucose disposal, and increasing physical activity prevents excess glucose from being stored as fat by raising energy expenditure (through muscle contractions) and promoting glucose uptake into muscles.

Human and rodent studies have shown that routine physical activity can improve liver histology and metabolic markers in MASLD. Both aerobic and resistance exercise regimens, whether accompanied by weight loss [278,279] or not [280,281,282], have been associated with improved cardiorespiratory fitness and reductions in intrahepatic triglyceride content compared to standard care. Adequate exercise—defined as >750 MET-minutes per week—has been shown to be 3.5 times more likely to result in clinically meaningful liver fat reduction compared to controls [278]. Although adherence is the most critical aspect of an exercise program, exercise intensity may also play a role in maximizing benefits. Different doses and intensities of exercise have been shown to positively modify hepatic steatosis [283,284,285]. Some studies report that higher-intensity exercise is associated with a lower prevalence of MASLD. High-intensity interval training (HIIT) has been shown to be a safe and effective strategy to improve liver health in MASLD patients. High-intensity exercise can reduce intrahepatic triglycerides and visceral fat in MASLD patients through improvements in energy expenditure, FAO, and reductions in visceral adipose tissue [286]. In one study, even the group with the lowest dose and intensity of exercise had reductions in visceral and hepatic fat regardless of weight loss, highlighting that any increase in activity can be beneficial [287].

### 5.2. Impacts of Exercise on Mitochondrial Function

#### 5.2.1. FAO and ROS

Reduced hepatic mitochondrial oxidative capacity has been shown to increase susceptibility to both hepatic steatosis and overall liver injury [132]. In a rodent model of 5 weeks of endurance treadmill training, there was no significant alteration in baseline liver mitochondrial function or OXPHOS activity. However, when hepatic toxicity was induced by salicylates, prior exercise training protected liver mitochondrial function, suggesting an induced resilience [288]. In contrast, a 4-week endurance training program in rats increased hepatic OXPHOS activity via complex I substrates but did not significantly alter liver mitochondrial biogenesis markers [289]. Additional studies have built on these findings, observing significant changes in specific mitochondrial ETC complexes in response to physical activity. One study reported exercise-induced increases in complex I, IV, and V activity, accompanied by elevated mitochondrial glutathione and no rise in ROS production [290]. In another study, 6 weeks of swimming exercise in rats decreased oxidative stress levels in hepatic mitochondria, attributed to increased antioxidant enzyme activity [291]. Together, these studies suggest that physical activity is effective in limiting ROS production and maintaining hepatic mitochondrial redox homeostasis [271]. In a study investigating the combined effects of metformin and physical activity in rats, exercise alone increased liver mitochondrial function despite the absence of weight loss, reinforcing the direct benefits of exercise on hepatic mitochondria [292].

#### 5.2.2. Biogenesis and Mitophagy

Mitochondrial biogenesis was first observed to increase in exercised muscle samples. This is logical, as the main regulator of this process—PGC-1α—is highly responsive to exercise stimuli [293]. During physical activity, skeletal muscle contractions cause fibers to produce myokines, which are released into the bloodstream and can affect non-contractile tissues. The liver is one of the main targets, and several exercise-induced myokines ultimately converge on activation of PGC-1α in hepatocytes. In support of this, increases in hepatic PGC-1α have been observed immediately following a single bout of endurance exercise, returning to baseline approximately 3 h afterward [294]. Improved mitochondrial biogenesis and turnover, as evidenced by transient rises in PGC-1α, may help explain some benefits of activity on hepatic function and are supported by several studies [295,296,297].

C1q-TNF-related protein 5 (CTRP5) is a myokine that promotes glucose uptake and fatty acid oxidation. CTRP5 may play a role in mediating exercise-induced improvements in MASLD. In HFD-fed mice, global knockout of CTRP5 resulted in reduced hepatic steatosis, suggesting that high levels of CTRP5 (as seen in sedentary obesity) may contribute to liver fat accumulation. Humans undergoing aerobic exercise have been shown to have reduced levels of plasma CTRP5, though changes in the periphery remain debated. An exercise-induced reduction in CTRP5 is thought to inhibit the mTORC1 complex, which, in turn, upregulates autophagy to clear abnormal liver mitochondria [298]. Exercise has also been shown to normalize HFD-induced changes in AMPK and mTORC1 phosphorylation, improving metabolic signaling [299]. Further research clarifying these effects in MASLD patients is needed to fully implicate a role for CTRP5 in this process.

Another myokine worth mentioning is irisin, which has been shown to induce AMPK signaling and subsequently reduce hepatocyte TG accumulation [300]. In a study of 10 individuals, high-intensity exercise increased circulating irisin by ~19% [301]. It is theorized that irisin can circulate and stimulate autophagy in hepatocytes, though there is debate over the downstream effects and actual significance of exercise-induced increases in irisin levels.

The intensity of exercise is also relevant to hepatic autophagy and lipid metabolism. At low exercise intensities, lipid oxidation predominates as a fuel source, whereas higher-intensity exercise relies more heavily on glucose metabolism [302,303,304]. In a study of rats undergoing treadmill training 5 days a week for 8 weeks at low, moderate, and high intensities, markers of increased autophagy were observed in the moderate- and high-intensity groups. Additionally, moderate- and high-intensity trained rats had decreased serum TG concentrations [304]. This finding suggests that exercise-induced hepatic autophagy may mediate improvements in liver lipid metabolism. Another study compared low-intensity exercise to a sedentary control and found acute increases in hepatic PGC-1α after exercise, with a return to basal levels ~3 h post-exercise [294]. The rise in PGC-1α indicates a transient boost in mitochondrial biogenesis and turnover, which may also contribute to improved hepatic function, as supported by multiple studies [295,296,297].

### 5.3. Ceramides During Physical Activity

Few studies have explored the impact of physical activity on ceramide levels in the liver in either humans or animal models. Our knowledge of this topic in skeletal muscle is more advanced and may provide insight into patterns in the liver. In general, higher skeletal muscle ceramide levels have been associated with VO2max indices of cardiorespiratory fitness, an important predictor of future health and disease risk [305]. Importantly, Plasma ceramide levels can be reduced through exercise training in obese individuals, with or without type 2 diabetes. For example, reductions in plasma ceramides C14:0, 16:0, 18:1, and 24:0 were observed after 12 weeks of aerobic training and after 16 weeks of moderate-intensity aerobic exercise in different studies [37,306]. In obese humans, a short-term exercise training program decreased skeletal muscle ceramide content, accompanied by enhanced glucose tolerance and insulin sensitivity. Furthermore, specific ceramide species in skeletal muscle were reduced (notably, C16:0, 16:1, 18:1, and 20:0) [307], a trend also observed across various rodent muscle types [308]. Interestingly, in longitudinal studies, exercise appears to have a limited effect on skeletal muscle ceramide concentration in healthy individuals; however, in individuals with elevated baseline ceramides due to obesity or type 2 diabetes, the same studies observed a decrease in skeletal muscle ceramide content with training [309]. The enhanced insulin sensitivity seen in endurance training studies of obese individuals may be partly attributed to improved mitochondrial function. More specifically, increased capacity for fatty acid uptake and oxidation in muscle leads to decreased lipid content and saturation in muscle tissue [309]. Improved insulin sensitivity can contribute to an exercise-mediated decline in ceramides by attenuating adipose lipolysis and inflammatory signaling, thereby reducing the substrate availability and enzymatic drive for de novo ceramide synthesis.

Longer bouts of exercise have also been studied. After trained athletes ran a half-marathon, total plasma ceramides significantly decreased. Inflammatory markers like IL-6 and CX3CL1 were transiently increased post-race, likely reflecting a physiological stress response rather than pathological damage [310]. The decrease in ceramides—coupled with their lack of correlation with the transient inflammation—supports a role for ceramides in beneficial training adaptations rather than harmful effects. In mice, it has been suggested that long-chain ceramide species can be reduced through endurance training [311]. Data from the half-marathon study revealed reduced C16:0 and C18:0 ceramides in athletes before the race but increased concentrations immediately after the race. This finding suggests that during the endurance event, there was reduced utilization of palmitate and stearate (precursors for ceramide synthesis), implicating these species as less-preferred fuel sources during exercise [310].

It appears that in stints of acute physical activity, plasma and muscle ceramide levels increase, while regular exercise is associated with decreases in these measurements [206]. It has been hypothesized that the short-term increase in ceramides during acute exercise could transiently inhibit insulin signaling and promote fatty acid oxidation, thereby helping to utilize lipids (in addition to glucose) as fuel [312]. The acute rise in ceramides may be explained by upstream increases in fatty acid mobilization during exercise. With regular training, normalization (or reduction) of ceramide levels may occur due to increased muscle fatty acid oxidation capacity, resulting in less substrate channeling into ceramide synthesis. Exercise may reduce ceramide production directly by lowering the availability of ceramide precursors (through improved fat combustion) and may accelerate ceramide degradation and clearance via upregulation of ceramidase gene expression [310]. Additional human studies are needed to determine how exercise alters hepatic and plasma ceramide concentrations.

### 5.4. The Synergistic Roles of Diet and Exercise in the Clinical Treatment of MASLD

**Figure 4:**
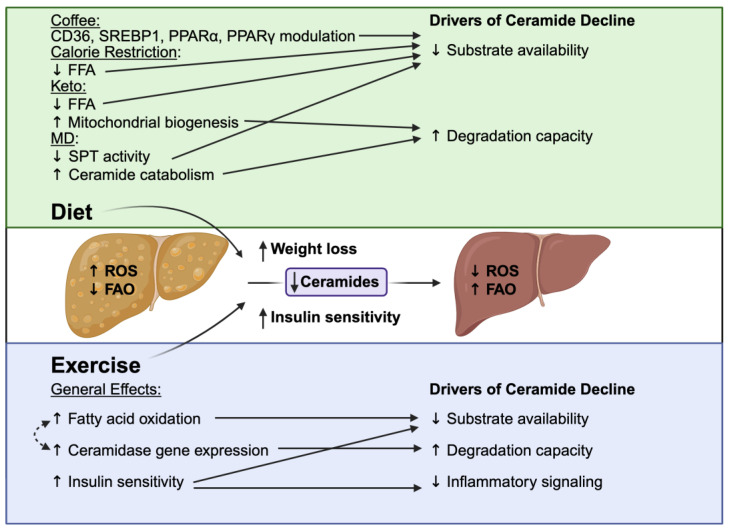
Ceramide-mediated effects of diet and exercise interventions on mitochondrial health in MASLD. SREBP1: sterol regulatory element-binding protein 1; PPAR: peroxisome proliferator-activated receptor; FFA: free fatty acid; SPT: serine palmitoyl transferase; ROS: reactive oxygen species; FAO: fatty acid oxidation. Created in BioRender. Available online: https://BioRender.com/ (accessed on 1 August 2025).

In clinical practice, diet and exercise are typically used together to facilitate weight loss in MASLD. The combined use of diet and exercise is recommended by major societies (e.g., the American Association for the Study of Liver Diseases and the American Gastroenterological Association) as the first-line therapy [219,313]. The effects of diet and exercise interventions on mitochondrial health and ceramides are summarized conceptually in Figure 4. In a rodent model of MASLD, both daily exercise and caloric restriction were able to attenuate the development of the disease. However, the exercise group may have experienced additional benefits, evidenced by greater improvement in markers of glucose homeostasis and hepatic mitochondrial function [314]. A 2020 meta-analysis suggested that physical activity combined with dietary treatment is associated with improvements in liver steatosis, liver enzymes, and serum lipid profiles [315].

In a recent study of humans with biopsy-proven MASH, subjects were randomized to either a control group (standard physician care) or a treatment group consisting of diet counseling plus HIIT exercise for 10 months [316]. Both groups reduced their total energy intake and showed improvements in hepatic insulin resistance and liver fat. However, the treatment group exhibited additional improvements in body weight, markers of liver injury, peripheral insulin sensitivity, and overall resolution of liver disease. The interaction between diet and exercise may be partially explained by the idea that exercise reroutes substrates away from hepatic tissue, easing its metabolic load. Mechanistically, skeletal muscle adapted to regular exercise may act as a sink for excess nutrients [316]. This could improve hepatic insulin sensitivity and fatty acid oxidation—both by meeting increased energy demands and by reducing fat storage—ultimately fostering regeneration of liver tissue. Overall, clinical guidelines should continue to recommend both diet and exercise as tools for weight loss in the prevention and treatment of MASLD and MASH.

Resmetirom, a thyroid hormone receptor-β agonist, is currently the only pharmacological treatment approved in the United States for adults with MASH. As described throughout this review, ceramides have several mechanistic avenues by which they may contribute to MASLD progression. Thus, a pharmacotherapeutic approach targeting ceramide synthesis could be of interest. Inhibition of ceramide-synthesizing enzymes, like SPT or CERS, could be considered, though more species-specific inhibition (e.g., targeting a particular CERS isoform) will likely be needed. As noted, certain ceramide species are more involved in MASLD pathogenesis (e.g., C16:0) compared to others that serve critical functions in normal membrane structure and cell signaling. There are ceramide synthase inhibitors, such as Fumonisin BB1 and FTY720, but they have non-specific activity toward ceramide synthases and either are too hepatotoxic or require further study regarding their effects on liver disease [317,318,319]. Fenretinide is a drug that inhibits dihydroceramide desaturase 1, the final enzyme in the ceramide synthesis pathway. Fenretinide prevents hepatic steatosis in animal models, but further studies in humans will be required to evaluate its efficacy and safety in MASLD [169,320]. Future research should clarify the details of ceramide interactions with their molecular targets, determine how different ceramide species contribute to overall liver health, and explore how ceramide metrics may be applied in the clinical setting. Plasma ceramide measurements are currently used in clinical practice primarily to predict cardiovascular event risk. Future studies examining established plasma ceramide scores (CERT1, CERT2, dScore, or SIC score) in relation to diet and exercise treatment in MASLD may help broaden diagnostic approaches and further explore associations between ceramides and MASLD.

## 6. Conclusions

MASLD is a progressive metabolic liver disease with potentially severe complications, such as cirrhosis. The prevalence of MASLD is growing worldwide, and effective pharmacologic therapeutics remain limited. Dietary modification and physical activity continue to be the primary recommendations for MASLD treatment and prevention. In this review, we have highlighted the central role of mitochondrial dysfunction in the development and progression of MASLD, with a focus on how ceramides mediate this process. Accumulation of ceramides within mitochondria is associated with key features of mitochondrial dysfunction in MASLD, including impaired fatty acid oxidation and increased oxidative stress. Our review of both animal models and human studies supports a potential role for ceramides in MASLD pathogenesis and suggests that ceramides are modulators responsive to dietary and exercise interventions. Future verification of mechanistic hypotheses in clinical studies is needed, but current findings underscore the potential of targeting ceramide metabolism and mitochondrial health as part of future therapeutic strategies for MASLD.

## Data Availability

Not applicable.
